# A mathematical-adapted model to analyze the characteristics for the mortality of COVID-19

**DOI:** 10.1038/s41598-022-09442-z

**Published:** 2022-03-31

**Authors:** Baobing Hao, Chengyou Liu, Yuhe Wang, Ninjun Zhu, Yong Ding, Jing Wu, Yu Wang, Fang Sun, Lixun Chen

**Affiliations:** 1 Department of Surgical Oncology, Jinling Hospital, School of Medicine, Nanjing University, Nanjing, 210000 Jiangsu Province China; 2 Department of Medical Engineering, Nanjing First Hospital, Nanjing Medical University, No. 68, Changle Road, Qinhuai District, Nanjing City, 210006 Jiangsu Province China; 3 Department of Neurology, Nanjing First Hospital, Nanjing Medical University, Nanjing, 210006 Jiangsu Province China; 4 Department of Mathematics and Computer, Nanjing Medical University, Nanjing, 210006 Jiangsu Province China; 5 Department of Medical Affairs, Nanjing First Hospital, Nanjing Medical University, 68 Changleroad, Nanjing, 210006 Jiangsu Province China; 6 Department of Critical Care Medicine, Nanjing First Hospital, Nanjing Medical University, No. 68, Changle Road, Qinhuai District, Nanjing City, 210006 Jiangsu Province China; 7 Department of Ophthalmology, Nanjing First Hospital, Nanjing Medical University, No. 68, Changle Road, Qinhuai District, Nanjing City, 210006 Jiangsu Province China

**Keywords:** Infectious diseases, Mathematics and computing

## Abstract

Severe acute respiratory syndrome coronavirus 2 (SARS-CoV-2) emerged in Wuhan, China, has led to the rapid development of Coronavirus disease 2019 (COVID-19) pandemic. COVID-19 represents a fatal disease with a great global public health importance. This study aims to develop a three-parameter Weibull mathematical model using continuous functions to represent discrete COVID-19 data. Subsequently, the model was applied to quantitatively analyze the characteristics for the mortality of COVID-19, including the age, sex, the length of symptom time to hospitalization time (SH), hospitalization date to death time (HD) and symptom time to death time time (SD) and others. A three-parameter mathematical model was developed by combining the reported cases in the Data Repository from the Center for Systems Science and Engineering at Johns Hopkins University and applied to estimate and analyze the characteristics for mortality of COVID-19. We found that the scale parameters of males and females were 5.85 and 5.45, respectively. Probability density functions in both males and females were negative skewness. 5% of male patients died under the age of 43.28 (44.37 for females), 50% died under 69.55 (73.25 for females), and 95% died under 86.59 (92.78 for females). The peak age of male death was 67.45 years, while that of female death was 71.10 years. The peak and median values of SH, HD and SD in male death were correspondingly 1.17, 5.18 and 10.30 days, and 4.29, 11.36 and 16.33 days, while those in female death were 1.19, 5.80 and 12.08 days, and 4.60, 12.44 and 17.67 days, respectively. The peak age of probability density in male and female deaths was 69.55 and 73.25 years, while the high point age of their mortality risk was 77.51 and 81.73 years, respectively. The mathematical model can fit and simulate the impact of various factors on IFR. From the simulation results of the model, we can intuitively find the IFR, peak age, average age and other information of each age. In terms of time factors, the mortality rate of the most susceptible population is not the highest, and the distribution of male patients is different from the distribution of females. This means that Self-protection and self-recovery in females against SARS-CoV-2 virus might be better than those of males. Males were more likely to be infected, more likely to be admitted to the ICU and more likely to die of COVID-19. Moreover, the infection fatality ration (IFR) of COVID-19 population was intrinsically linked to the infection age. Public health measures to protect vulnerable sex and age groups might be a simple and effective way to reduce IFR.

## Introduction

China was the first country to recognize the Coronavirus Disease 2019 (COVID-19), earlier termed as the 2019 Novel Coronavirus (2019-nCoV). Later, it was called SARS Cov-2, leading to severe acute respiratory syndrome. The main symptoms include fever, dry cough, fatigue, sore throat, loss of taste or smell, headache, diarrhea, difficulty of breathing, and/or chest pain. Many individuals were continuously and reportedly getting sick after being exposed from the virus. Due to its highly infective nature, the contagious disease spread across all Chinese provinces after almost a month. Concurrent with the nationwide spread, it also reached outside mainland China just after 13 day. Despite the great efforts made by China to contain the disease, it spread rapidly all over the world, causing an ongoing pandemic^1^.

In January 2020, a study published in the Lancet indicated that COVID-19 symptoms first appeared on December 1, 2019^2^. Many scholars believe that the virus originated in animals and spread by spillover infection^3,4^. Professor Zhong Nanshan and the World Health Organization (WHO) confirmed human-to-human transmission of the virus on 20 January 2020^5^. According to official data from China, most cases of SARS-CoV-2 human-to-human transmission linked to the South China Seafood Wholesale Market^6^. In the early stages of the COVID-19 outbreak, the number of people diagnosed with COVID-19 doubled in about 7.5 days^7^. In January 2020, during the Chinese New Year, the rate of population migration increased dramatically, and SARS-CoV-2 began spreading to other Chinese cities^8^. By that time, official Chinese data indicated that 6,174 people in China had developed COVID-19 symptoms, but more suspected cases may have been infected^9,10^. The personal protective equipment (PPE) was strongly recommended for health workers, according to a report published in the Lancet on 24 January 2020, citing the characteristics of human-to-human transmission of COVID-19^11,12^. On January 30, 2020, when the WHO listed the COVID-19 epidemic as a Public Health Emergency of International Concern (PHEIC), the spread of SARS-CoV-2 increased nearly 200 times^13,14^. On January 31, 2020, SARS-CoV-2 has spread to Italy and the first confirmed case of COVID-19 was announced^15^. As of March 13, 2020, WHO considered Europe to be the active epicenter of the pandemic^16^. On March 19, 2020, Italy became the country with the most COVID-19 deaths^17^. Up to March 26, the United States has replaced Italy and China as the country with the most confirmed cases of COVID-19^18^.

According to the National Health Commission of China, so far, COVID-19 has caused a total of 263,028,578 confirmed cases and 5,233,966 deaths worldwide. The mortality rate of confirmed cases in China was 5.2% (6697/127,938). Meanwhile, the mortality rate was 2.0% (5,227,269/262,900,640) among cases outside China. COVID-19 is highly infectious with a relatively high mortality rate. However, the messages obtainable in Internet reports and published studies are speedily increasing. In order to help medical workers around the world to better deal with COVID-19, we reviewed the relevant references and provided a general scenario mathematical model for relevant researchers, so as to prepare for the widespread epidemic of COVID-19.

## Methods

### Three-parameter Weibull data distribution model

Probability theory is the branch of mathematics concerned with probability. Although there are several different probability interpretations, probability theory adopts the concept in a rigorous mathematical manner through a set of axioms. It is a mathematical description of a random phenomenon in terms of its sample space and event probability (subsets of the sample space)^19^.

Survival analysis is a branch of probability theory, which is used for analyzing the expected duration of time until one or more events happen, such as death in biological organisms and failure in biological systems. This topic is called reliability theory or reliability analysis, and event history analysis in sociology. Survival analysis attempts to answer certain questions, such as what is the survival proportion of the population after a certain time? How quickly will those who survive die or fail? How does a particular situation or feature increase or decrease the probability of survival?

In probability theory and statistics, the Weibull distribution is a continuous probability distribution. It is named after Swedish mathematician Waloddi Weibull, who described it in detail in 1951, although it was first identified by Fréchet (1927) and first applied by Rosin & Rammler (1933) to describe a particle size distribution^20^.

From the perspective of probability theory and statistics, the probability density function of a three-parameter Weibull data distribution model random variable is as follows:

1 $$f\left( {t,\lambda ,k,t_{0} } \right) = \left\{ {\begin{array}{*{20}l} {\frac{k}{\lambda }\left( {\frac{{t - t_{0} }}{\lambda }} \right)^{k - 1} e^{{ - \left( {\frac{{t - t_{0} }}{\lambda }} \right)^{k} }} } \hfill & {t \ge t_{0} } \hfill \\ 0 \hfill & {t < t_{0} } \hfill \\ \end{array} } \right.$$

$k > 0$ is the shape parameter. $t_{0} \ge 0$ is the location parameter. $\lambda > 0$ is the scale parameter of the function. Its complementary cumulative distribution function is a stretched exponential function. The Weibull distribution is related to the number of other probability distributions; In particular, it interpolates between the exponential distribution ($k = 1$) and the Rayleigh distribution ($k = 2, \lambda = \sqrt 2 \sigma )$.

### Cumulative distribution and reliability function

The reliability function of the Weibull data distribution model reflects the availability of the remaining lives of biological organisms. The definition of reliability function in reliability theory engineering is defined as the specific residual viability of biological organisms under specified conditions and time points. The cumulative density distribution function and reliability function can be calculated by Formula 1.

2 $$\left\{ {\begin{array}{*{20}l} {F\left( {t,\lambda ,k,t_{0} } \right) = \mathop \smallint \limits_{0}^{t} f\left( {\mu ,\lambda ,k,t_{0} } \right)d\mu = e^{{ - \left( {\frac{{t - t_{0} }}{\lambda }} \right)^{k} }} } \hfill \\ {R\left( {t,\lambda ,k,t_{0} } \right) = 1 - F\left( {t,\lambda ,k,t_{0} } \right) = 1 - e^{{ - \left( {\frac{{t - t_{0} }}{\lambda }} \right)^{k} }} } \hfill \\ \end{array} } \right.$$

**Figure 1:**
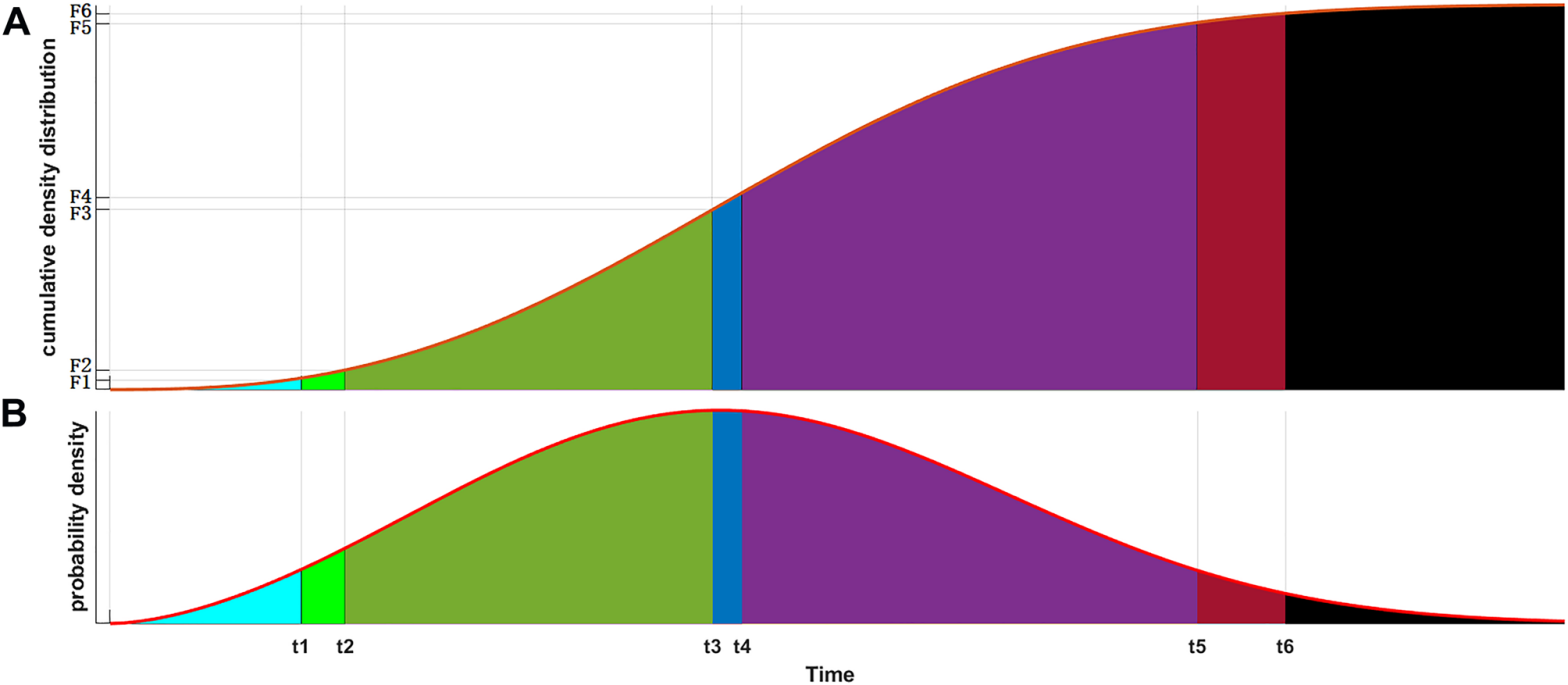
Calculation results of important time nodes. (**A**) Cumulative distribution function distribution. The abscissa is time. F1 = 2.5%, F2 = 5%, F4 = 50%, F5 = 95%, F6 = 97.5% and F3 is the F.peak, which can be calculated by Figure B. (**B**) Probability density function distribution, in which t1 = T.lower 97.5% CI, t2 = T.lower 95% CI, t3 = T.peak, t4 = T.median, t5 = T.upper 95% CI and t6 = T.upper 97.5% CI.

According to the probability theory, important time nodes, such as “T.lower 95% CI” and “T.upper 95% CI”, “T.lower 97.5% CI” and T.upper 97.5% CI”, “T.peak” and “T. median” (Fig. 1), can be calculated by the cumulative distribution function (Fig. 1).

3 $$T = \left[ {\begin{array}{*{20}c} {{\text{T}}.{\text{lower }}97.5{\text{\% CI}}} \\ {{\text{T}}.{\text{lower }}95{\text{\% CI}}} \\ {\begin{array}{*{20}c} {{\text{T}}.{\text{peak}}} \\ {{\text{T}}.{\text{ median}}} \\ {\begin{array}{*{20}c} {{\text{T}}.{\text{upper }}95{\text{\% CI}}} \\ {{\text{T}}.{\text{upper }}97.5{\text{\% CI}}} \\ \end{array} } \\ \end{array} } \\ \end{array} } \right] = t_{0} - \lambda *\ln \left( {\left[ {\begin{array}{*{20}c} {{\text{F}}.{\text{lower }}97.5{\text{\% CI}}} \\ {{\text{F}}.{\text{lower }}95{\text{\% CI}}} \\ {\begin{array}{*{20}c} {{\text{f}}.{\text{peak}}} \\ {{\text{f}}.{\text{ median}}} \\ {\begin{array}{*{20}c} {{\text{F}}.{\text{upper }}95{\text{\% CI}}} \\ {{\text{F}}.{\text{upper }}97.5{\text{\% CI}}} \\ \end{array} } \\ \end{array} } \\ \end{array} } \right]} \right)^{\frac{1}{k}}$$

### Ethics approval and consent to participate

Not applicable.

### Patient consent for publication

Not applicable.

## Estimation of Weibull data distribution model parameter

There are many methods to estimate the parameters of mathematical models, especially probability mathematical models. Commonly used methods include Gaussian estimation method, graphical method, least square method, maximum likelihood estimation method, etc.^21–24^ In this paper, we transformed the three-parameter Weibull data distribution model and used the maximum likelihood estimation method to estimate the model parameters.

Let $\psi = \left( {\lambda ,k,t_{0} } \right)$. We used the logarithmic function to transform the three-parameter Weibull data distribution model. Let $M = \left\{ {t_{1} ,t_{2} ,t_{3} , \ldots t_{n} } \right\}$. The model parameters were estimated as follows:

4 $$\left\{ {\begin{array}{*{20}l} {\frac{{\partial \left[ {\ln \left[ {L\left( {\psi {|}M} \right)} \right]} \right]}}{\partial k} = \mathop \sum \limits_{i = 1}^{n} \left[ {\frac{1}{k} + {\text{ln}}\left( {t_{i} - t_{0} } \right) - ln\lambda - \left( {\frac{{t_{i} - t_{0} }}{\lambda }} \right)^{k} {\text{ln}}\left( {\frac{{t_{i} - t_{0} }}{\lambda }} \right)} \right] = 0} \hfill \\ {\frac{{\partial \left[ {\ln \left[ {L\left( {\psi {|}M} \right)} \right]} \right]}}{\partial \lambda } = \mathop \sum \limits_{i = 1}^{n} \left[ { - \frac{k}{\lambda } + \frac{{k\left( {t_{i} - t_{0} } \right)^{k} }}{{\lambda^{k + 1} }}} \right] = 0} \hfill \\ {\frac{{\partial \left[ {\ln \left[ {L\left( {\psi {|}M} \right)} \right]} \right]}}{{\partial t_{0} }} = \mathop \sum \limits_{i = 1}^{n} \left[ { - \frac{k - 1}{{t_{i} - t_{0} }} + \frac{{k\left( {t_{i} - t_{0} } \right)^{k - 1} }}{{\lambda^{k} }}} \right] = 0 } \hfill \\ \end{array} } \right.$$

### Source of the COVID-19

The complete COVID-19 data set is a collection of COVID-19 data maintained by COVID-19 Data Repository by the CSSE at Johns Hopkins University (JHU) (https://github.com/CSSEGISandData/COVID-19). These data updated daily include numbers of confirmed cases, deaths, hospitalized cases, and testing cases, as well as other variables of potential interests, such as the age, sex, duration of symptoms, date of hospitalization, time of admission to ICU care, time of death, etc. The case & death data set is updated daily. The number of cases or deaths reported by any institution, including JHU, WHO, European Centre for Disease Prevention and Control (ECDC) and others, on a given day does not necessarily represent the actual number on that date.

### Statement confirmation

All collected data were disclosed by the CSSE and validated by manual verification. The CSSE approved the waiver of informed consent. All data and materials are fully available without restriction. This study did not infringe on patient’s privacy or health, and was performed according to the Declaration of Helsinki.

## Result

### General characteristics and quality of COVID-19 cases

**Figure 2:**
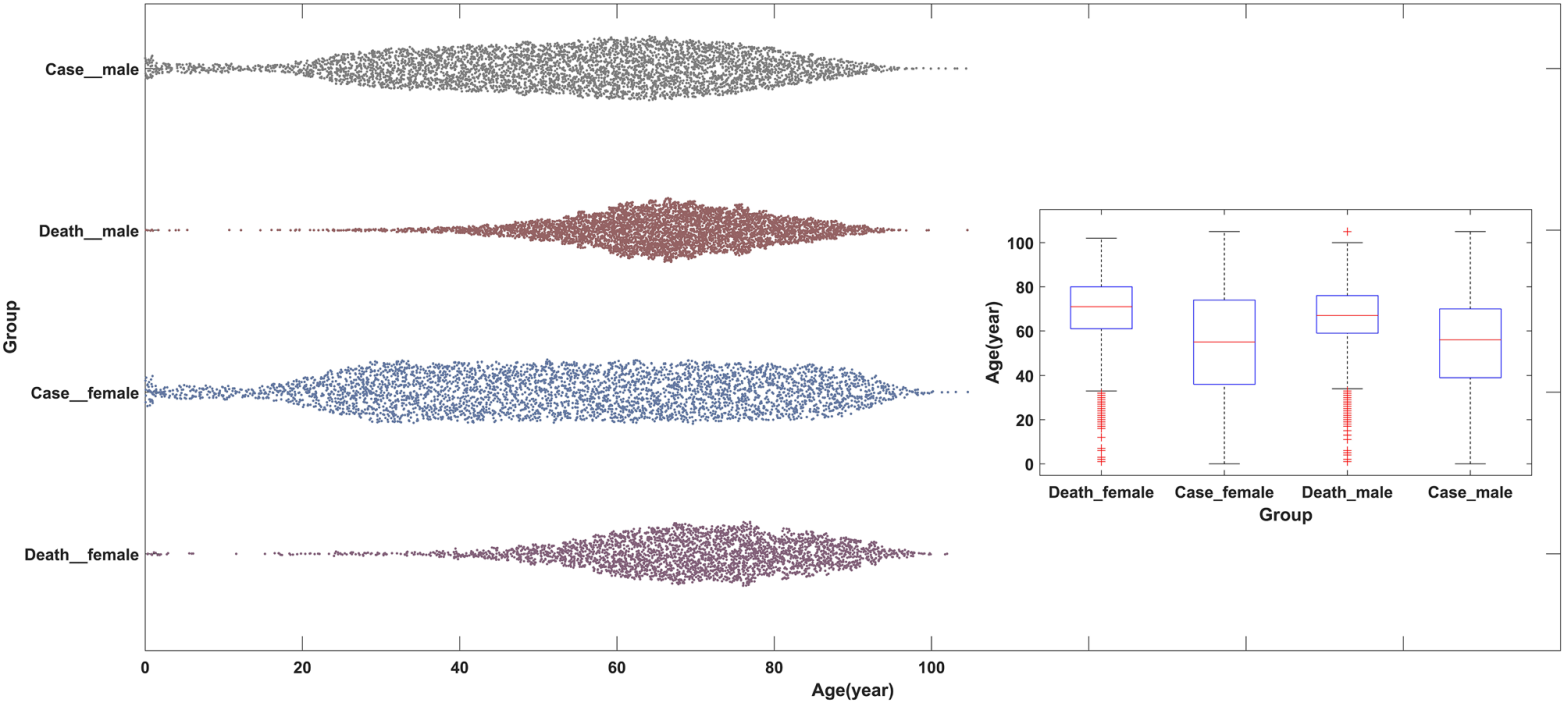
General characteristics and quality of COVID-19 cases. The scatter diagrams from the top to bottom were male cases who were infected with SARS-CoV-2 and hospitalized, male cases who were hospitalized and died, female cases who were infected with SARS-CoV-2 and hospitalized, and female cases who were hospitalized and died. The box plots from left to right corresponded to the scatter diagrams from the top to bottom.

The general characteristics and quality of COVID-19 about patients are shown in Fig. 2 and Table 1. As of the time of writing the manuscript, there were 88,911 hospitalizations caused by SARS-CoV-2 reported in CSSE, 14,529 patients in ICU and 8873 deaths in hospitals. Among the patients of COVID-19, 54.17% (48,162/88,911) were male, and 45.83% (40,749/88,911) were female. 59.01% (20,913/35,442) male cases and 40.99% (14,529/35,442) female cases who were sent to ICU, and 63.34% (5620/8873) male deaths and 36.66% (3253/8873) female deaths. At the same level, the proportion of male patients gradually increased (95% CI, *P* < 0.01). The impact of COVID-19 on the mortality differed from men and women. Among hospitalized cases, the mortality rate for males and females was 11.7% (5620/48,162) and 8.0% (3253/40,749), respectively, which was significantly higher in males with the ratio was 1.73:1 (95%CI, *P* < 0.01). The death age of males and females was normally distributed. Median death age was 66.5 years for males and 69.7 years for females. Variance age was 13.4 years for males and 15.2 years for females (95%CI, *P* < 0.01). 44.13% of hospitalizations occurred in patients over 70 years of age and 75.78% of deaths within that age bracket. The age distribution of hospitalized male and female cases tended to be uniform distributed, the median age of which was 54.2 years for males and 54.4 years for females. Meanwhile, the variance age was 20.8 years for males and 23.3 years for females (95%CI, *P* = 0.45).

**Table 1:** Mathematical statistics calculation results by age and sex based on cases from CSSE.

Age	Infected people who are hospitalized			Hospitalized people who go to Intensive Care Unit			Hospitalized people who die		
	Male	Female	Total	Male	Female	Total	Male	Female	Total
0–19	2553 (2.87)	2583 (2.91)	5136 (5.78)	1292 (3.65)	1086 (3.06)	2378 (6.71)	17 (0.19)	26 (0.29)	43 (0.48)
20–29	3773 (4.24)	4015 (4.52)	7788 (8.76)	708 (2.00)	578 (1.63)	1286 (3.63)	46 (0.52)	34 (0.38)	80 (0.90)
30–39	5863 (6.59)	5480 (6.16)	11,343 (12.76)	1036 (2.92)	663 (1.87)	1699 (4.79)	123 (1.39)	54 (0.61)	177 (1.99)
40–49	6847 (7.70)	5378 (6.05)	12,225 (13.75)	1848 (5.21)	1010 (2.85)	2858 (8.06)	367 (4.14)	176 (1.98)	543 (6.12)
50–59	7758 (8.73)	5425 (6.10)	13,183 (14.8)	3189 (8.99)	1640 (4.62)	4829 (13.6)	906 (10.2)	400 (4.50)	1306 (14.7)
60–69	8785 (9.88)	5457 (6.14)	14,242 (16.02)	4996 (14.10)	2585 (7.29)	7581 (21.39)	1769 (19.94)	812 (9.15)	2581 (29.09)
70–79	7321 (8.23)	5357 (6.03)	12,678 (14.26)	4609 (13.00)	3072 (8.67)	7681 (21.67)	1493 (16.83)	872 (9.83)	2365 (26.65)
80–89	4262 (4.79)	4867 (5.47)	9129 (10.27)	2689 (7.59)	2858 (8.06)	5547 (15.65)	765 (8.62)	637 (7.18)	1402 (15.80)
90 +	1000 (1.12)	2187 (2.46)	3187 (3.58)	546 (1.54)	1037 (2.93)	1583 (4.47)	134 (1.51)	242 (2.73)	376 (4.24)
Total	48,162 (54.17)	40,749 (45.83)	88,911 (100.00)	20,913 (59.01)	14,529 (40.99)	35,442 (100.00)	5620 (63.34)	3253 (36.66)	8873 (100.00)

In Table 1, patients infected with SARS-CoV-2 were divided into six groups according to the sex, outcome and important time nodes. They were male/female cases who were infected with SARS-CoV-2 and hospitalized, male/female cases who were hospitalized and sent to ICU, and male/female cases who were hospitalized and died. According to age composition shown in Table 1, patients infected with SARS-CoV-2 were divided into 9 groups, including 0–19 years old group, 20–29 years old group, 30–39 years old group, 40–49 years old group, 50–59 years old group, 60–69 years old group, 70–79 years old group, 80–89 years old group and 90 + years old group.

### Model calculations and fitting by age and sex based on deaths in hospital

**Figure 3:**
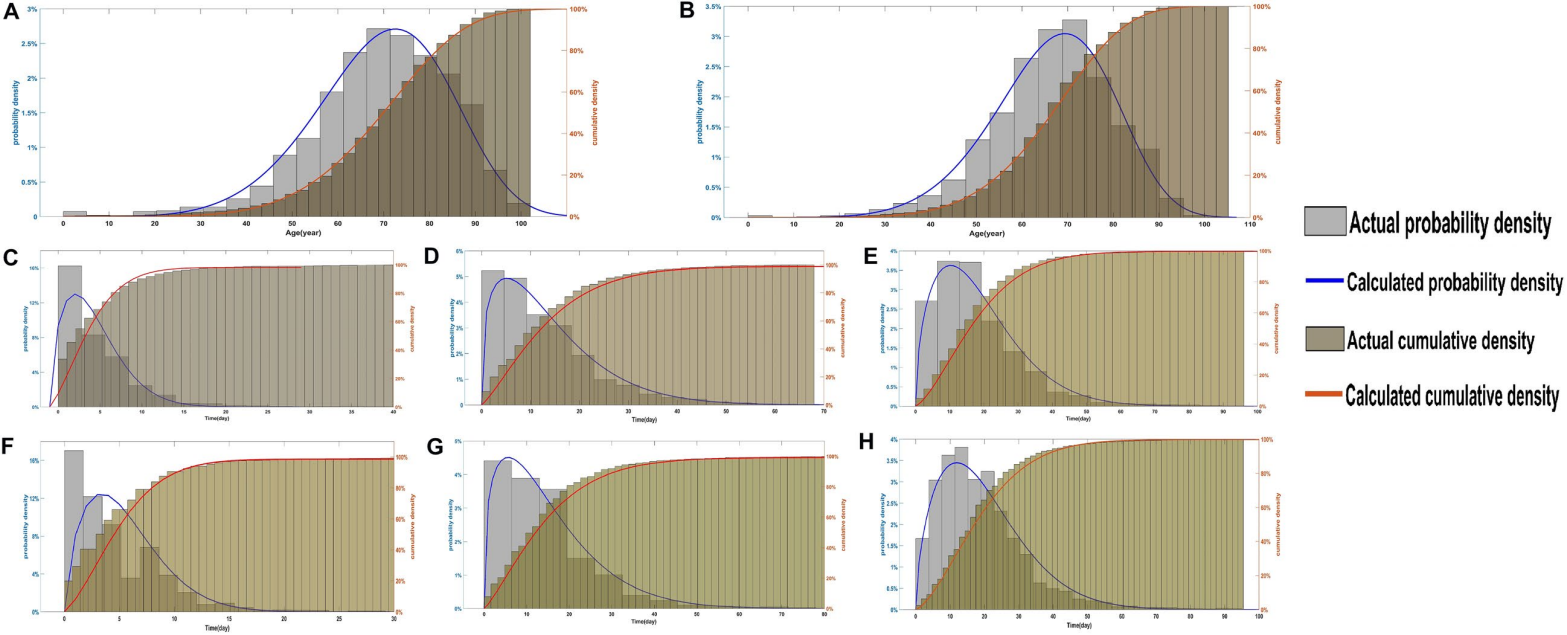
The results of model calculation used the COVID-19 data set. The gray histogram represented the actual probability density. The khaki histogram indicated the actual cumulative density. The blue curve represented the calculated probability density, and the yellow curve represented the calculated cumulative density. The gray histogram and blue curve were shown in principal coordinates, and the khaki histogram and yellow curve were shown in secondary coordinates. The results of model calculation used the (**A**) male and (**B**) female death data set. The results included the age at the death of male and female actual/calculated probability density and actual/ calculated cumulative density. The models of (**C**) SH, (**D**) HD and (**E**) SD time for males, who were hospitalized and died, including SH, HD, SD time actual/calculated probability density and actual/ calculated cumulative density. The models of (**F**) SH, (**G**) HD and (**H**) SD time for females, who were hospitalized and died, included SH, HD, SD time actual/calculated probability density and actual/ calculated cumulative density.

The parameters of the three-parameter Weibull data distribution model were estimated by selecting the complete COVID-19 data set. The main variables were the age, sex, symptom time, hospitalization date, time of admission to ICU care, and death time. The calculation results of model parameters were shown in Table 2 and Fig. 3. Through the calculation results, for the age parameter, we found that the scale parameters of males and females who were infected and died, were 5.85 and 5.45, respectively. It is suggested that the probability density functions of the two groups were left-skewed curves, and females were more left-skewed (distribution with negative skewness) than males. At the same time, the location parameters of the two groups were 0.15 and 0.73, respectively, and the shape parameters of which were 71.67 and 75.29, respectively. For males, 63.2% died from 0.15 to 71.67 years, and for females, 63.2% died from 0.73 to 75.29 years.

**Table2:** The calculation results of model parameters and import time nodes.

Group			Model parameter			The important time nodes in the model					
			K	t0	$\Lambda$	t1	t2	t3	t4	t5	t6
Age groups	Male		71.67	0.15	5.85	38.38	43.28	69.55	67.45	86.59	89.72
	Female		75.29	0.73	5.45	39.07	44.37	73.25	71.10	92.78	96.36
Time nodes groups	Male	SH	5.81	0.04	1.17	0.30	0.50	1.17	4.29	14.89	17.77
		HD	14.89	0.08	1.32	1.00	1.65	5.18	11.36	34.27	40.11
		SD	20.66	0.09	1.52	1.93	3.02	10.30	16.33	42.62	48.86
	Female	SH	6.24	0.05	1.16	0.32	0.54	1.19	4.60	16.12	19.28
		HD	16.28	0.08	1.33	1.11	1.83	5.80	12.44	37.23	43.53
		SD	22.07	0.11	1.6	2.33	3.56	12.08	17.67	43.93	50.02

SH: the length of the symptom time to hospitalization date; HD: the length of the hospitalization date to death time; SD: the length of the symptom time to death time. t0: the location parameter; k: the shape parameter; λ: the scale parameter. t1 = T.lower 97.5% CI, t2 = T.lower 95% CI, t3 = T.peak, t4 = T.median, t5 = T.upper 95% CI and t6 = T.upper 97.5% CI.

Similarly, we calculated the model parameters using the symptom time, hospitalization date and death time. The results were shown in Table 2 and Fig. 3. In this paper, we defined the length of symptom time to hospitalization date as SH time, the length of hospitalization date to death time as HD time and the length of symptom time to death time as SD time. Through the model calculation, we found that the scale parameters of SH, HD and SD time for male patients were 1.17, 1.32 and 1.52, respectively. The scale parameters of SH, HD and SD time for female patients were 1.16, 1.33 and 1.60. In other words, all probability density curves were right-skewed (distribution with negative skewness). The shape parameters of SH, HD and SD for males were 5.81, 14.89, and 20.66, respectively, which were 6.24, 16.28 and 22.07 for females, respectively. Among death cases, 63.2% of male patients were hospitalized within 5.81 days (6.24 days of female patients) after symptom confirmation and died within 20.66 days (22.07 days of female patients).

As shown in Table 2, 2.5% males were hospitalized and died under 38.38 years (39.07 years of female patients), and 5% males were died under 43.28 years (44.37 years of female patients). Likewise, 5% males were died above the age of 86.59 years (92.78 years of female patients), and 2.5% males were died above the age of 89.72 years (96.36 years of female patients). The median age of males who died was 69.55 years (73.25 years of female patients). The mortality rate for males reached peak at 67.45 years (71.10 of females) SH, HD, SD time for males and females showed the same regular pattern (Table 2).

### Influence of age risk factors on mortality

**Figure 4:**
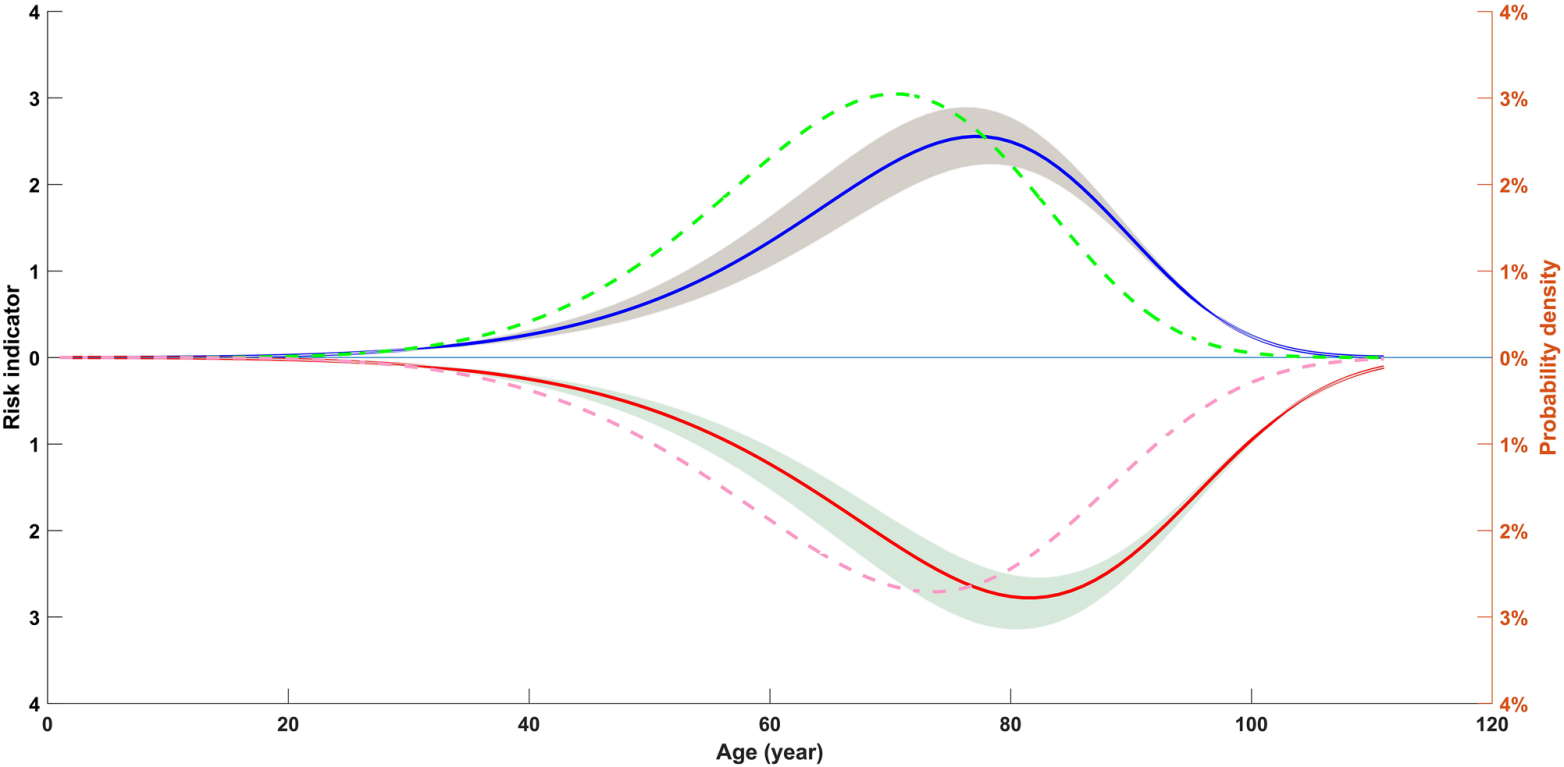
Influence of age risk factors on mortality. The green dotted line indicated the probability density of male death, and pink dotted line indicated female death. The blue line denoted the regression estimate of male mortality risk as a function of age, and red line denoted female mortality risk. The shaded region depicted the 95% confidence interval of the estimation.

As shown in Fig. 4, it was evident that the mortality risk of COVID-19 for the elderly was many times higher than of the young. In fact, most COVID-19 deaths were elderly. In this article, 71.4% of male deaths and 76.6% of female deaths were older than 60 years old. Under the age of 40, the mortality risk of COVID-19 was lower. However, the mortality risk of deaths after 40 years of age increased rapidly, which reached highest at the age of 77.51 years. In addition, we found some interesting phenomena as follows. The highest mortality risk of female deaths was at the age of 81.73 years, while for male deaths was at the age of 77.51 years. For the influence of age factor, there was a significant difference between males and females on the mortality risk. In other words, the mortality risk for females was significantly later than that of males. For male deaths, although the peak age of death probability density was 69.55 years, the highest point age of mortality risk was 77.51 years. Similarly, the peak age of death probability density for females was 73.25 years, but the maximum point age of mortality risk was 81.73 years.

## Discussion and conclusion

A review of COVID-19 epidemiological data showed that there were gender differences in COVID-19 disease. Compared with other countries, male COVID-19 in China and Italy have higher mortality rates^25–27^. According to official Chinese data, the mortality rate for men was 2.8% and women was 1.7%^28^. Male lifestyle, such as smoking and alcohol consumption, may be the main factor behind the difference in mortality rates between men and women with COVID-19 in China, according to an epidemiological review. From the immunological point of view, bad habits such as smoking and drinking may be the main causes of hypertension, cardiovascular and lung cancer, and may also be the main factors leading to higher male mortality^29,30^. Official data in Europe have similar conclusions, men were more likely to be infected (57%), and also more likely to die (72%)^31^.

There were many key indicators, such as mortality rate (MR), case fatality rate (CFR) and infection rate (IFR), etc., which can be used to judge the severity of COVID-19^32^. Among these indicators, IFR was the most widely used and was used for explosive infectious diseases. IFR represented the percentage of deaths of all infected people, including those who died asymptomatic and undiagnosed. However, these key indicators were limited by differences in time, quality of the health care system, age and gender and other factors.

At the early stage of COVID-19 outbreak, IFR reported by WHO was below 1%^33,34^. In August 2020, the chief scientist of WHO pointed that if the results of broad serology testing in Europe were included in the study, the IFR estimate was evaluated to converge between about 0.5% and 1%^35^. In September 2020, the U.S. Centers for Disease Control and Prevention conducted the first age-specific IFR study for public health programs^36^. In December 2020, a review and meta-analysis displayed that IFR was converge between 0.5 and 1% in many countries (Portugal, France, etc.), more than 2% in Italy, and between 1 and 2% in others (UK, Spain, etc.)^37^. The study also pointed that the differences in IFR indirectly reflected the differences in disease infection rates among different age groups. The IFR value of younger adults and children was very low (e.g., 0.002% at age 10 and 0.01% at age 25). However, with the increase of age, IFR increased faster. For example, at the age of 55, the IFR was 0.4% (e.g., 1.4% at age 65 and 15% at age 85). These results were also highlighted in a December 2020 report issued by the WHO^38^.

In this article, we proposed a three-parameter Weibull model to fit the COVID-19 data set, including age, sex, symptom time, hospitalization date, time of admission to ICU care, death time. At the same time, we could intuitively use continuous functions to qualitatively expressed continuous data. Overall, males infected by SARS-CoV-2 were more dangerous than females. Male-to-female ratios of hospitalized patients, ICU patients, and died patients were 1.18:1(48,162:40,749), 1.22:1 (20,913:14,529) and 1.73:1(5620:3253). Through calculation, for the patients who died from COVID-19, we found that the scale parameters of males and females were 5.85 and 5.45, location parameters were 0.15 and 0.73, and shape parameters were 71.67 and 75.29. Both probability density functions of males and females were negative skewness distributions, and females were more left-skewed than males. Further calculations indicated that 5% of males died under the age of 43.28 (44.37 for females), 50% died under 69.55 (73.25 for females), and 95% died under 86.59 (92.78 for females). In addition, the peak age of death in males was 67.45 years old, while that of females was 71.10 years old. In fact, the ages of male and female death were 66.5 ± 13.4 and 69.7 ± 15.2 years. From these results, we found that males were more likely to be infected, more likely to be admitted to the ICU and more likely to die, and the death age was generally younger than females. Those conclusions were similar to the comments of many scholars in the world^39,40^. These findings suggested that it might be attributable to work style choices such as heavy workload, dangerous working environment, lifestyle choices such as smoking and drinking alcohol. In the early stages of the pandemic, the observation that males were more susceptible to COVID-19 was speculated to be due to gender differences in social behavior. Males were more likely to downplay the risk of COVID-19, ignoring preventive advices such as social distancing and wearing masks, and participating in mid-high-risk activities such as public gatherings.

In addition, we obtained some unexpected phenomena in this study. For example, the peak value of SH for male deaths was 1.17 days (1.19 days for females), and the median value was 4.29 days (4.60 days for females). Similarly, the peak values for males and females were 5.18 and 5.80 days, and the median values were 11.36 and 12.44 days. The peak values for males and females were 10.30 and 12.08 days, and the median values were 16.33 and 17.67 days, respectively. Both the peak values and median values of SH, HD and SD time, female were longer than those of males. However, the mortality rate of female was significantly lower than that of male, suggesting after being infected with the SARS-CoV-2 virus, females received treatment more later and had a longer struggle with the SARS-CoV-2 virus, but had a higher probability of survival than that of males. It is also indicated that self-protection and self-recovery against SARS-CoV-2 virus in females might be better than those of males. In fact, it was consistent with the situation observed in previous SARS-CoV and MERS-CoV (or other large-scale infectious diseases) infections. Moreover, in the COVID-19 data set, we found that for both male and female deaths, the peak risk age of death data was greater than the peak age of the probability density of deaths, and the peak risk age of males was smaller than that of females. This showed that the population IFR was intrinsically linked to the specific age group of infection. Therefore, in order to reduce the overall IFR, public health measures to protect vulnerable sex and age groups may be a simple and effective measure. For example, when the amount of vaccine is severely insufficient, giving priority to the distribution of vaccines according to sex and age groups may be the most important public health measure.

However, general behaviour (habits) and biological factors (immune response) can determine the consequences of COVID-19^41^. Many of those who die of COVID-19 have pre-existing conditions, including hypertension, diabetes mellitus, and cardiovascular disease, etc.^42^ According to the CDC report, the most common comorbidities of COVID-19 are respiratory syndrome, including moderate or severe asthma, pre-existing COPD, pulmonary fibrosis, cystic fibrosis^43^. When someone with existing comorbidities problems is infected with COVID-19, they might be at greater risk for severe symptoms. When completing this continuous mathematical model, we realized the limitations of this study. This model can be used to explain the general continuity problems, including exponential distribution, Rayleigh distribution, normal distribution, partial normal distribution, average distribution, but cannot be used to explain the discrete (comorbidities) problems. This question will be the focus of our research.

## Supplementary Information

Supplementary Information 1.

Supplementary Information 2.

## Data Availability

The complete COVID-19 data set is a collection of the COVID-19 data maintained by COVID-19 Data Repository by the Center for Systems Science and Engineering (CSSE) at Johns Hopkins University (https://github.com/CSSEGISandData/COVID-19). All data and materials are fully available without restriction.
